# Incorporation of the health care system in the west

**Published:** 2015-09-30

**Authors:** Floro Hermes Gomez Pineda

**Affiliations:** Social and Public Health and Community Medicine Area, Faculty of Health Sciences, Universidad Libre, Cali, Colombia

**Keywords:** Health care, hospitals accumulation, medical practices, hospital wards, medicalization, sanitizing machine, the West

## Abstract

A reflection is made, from an interpretative perspective, on the historical evolution of health care in the West. It starts from the moment that this became a way to intervene the sick and an instrument for healing diseases, focusing on original documents and written sources which account for results of historical research, which range from XV century until today. To do this, it tries to understand the health care as an ideographic body of knowledge consisting of five pieces of a puzzle composed by: the state policy of hospitals accumulation implemented in Spain, the accumulation of medical practices in what is currently Germany, the hospital wards in England, the nosological rationality in France, and the US sanitizing machine; all these movements as producers of closely linked health care developments, that are nothing more than collective actions regulated by social norms around health.

## Abstract (es)

Se hace una reflexión, desde una perspectiva interpretativa, sobre la evolución histórica de la atención de la salud en Occidente. Se parte desde el momento en que esta se transformó en una manera de intervenir a los enfermos y en un instrumento para la curación de las enfermedades, centrándose en documentos y fuentes originales escritas que dan cuenta de resultados de investigaciones históricas, las cuales abarcan desde el siglo XV hasta hoy. Para ello, trata de comprender la atención de la salud como un cuerpo ideográfico de conocimientos, integrado por cinco piezas de un rompecabezas compuesto por: La política estatal de acumulación de hospitales implementada en España, la acumulación de las prácticas médicas en lo que hoy es Alemania, el hospital de pabellones en Inglaterra, la racionalidad nosológica en Francia y la máquina sanitaria en Estados Unidos; todos estos movimientos como productores de unos desarrollos asistenciales sanitarios estrechamente vinculados, que no son otra cosa que acciones colectivas reguladas por normas sociales en torno a la salud.

## Introduction

This article is a reflection, from an interpretative perspective, about the results of various investigations that comprise one of the components of the health system in the West, namely: the system of health care. Therefore, it is not about the other components: the steering systems (direction, regularization and control), financing, resource development and health assurance.

On the other hand, it streamlines the health care system, which is understood as an ideographic body of knowledge, articulating the many different strands of the care fabric, which original sources show from the fifteenth century to the present day to show a progressive historical development of incorporation, regulated by social norms charged with meanings of various kinds of the way how it has been incrementally stepping the intervention on the sick, and how the health care has been transformed into an instrument for healing various diseases.

In this regard, the question arises: when did it begin to be incorporated into the health system the idea of ​​a health care system as a way of intervention on the sick and as a tool for healing diseases?

This question starts from a premise: to understand that the historical evolution of the health care in the West forms one body, and that the developments of incorporation for each geographic area are all pieces of the same puzzle. Likewise, it is exposed the result of research for each geographical area, serving a previous order (when possible), and partial interpretations are proposed, independently of a final interpretation, in specific areas of health care.

## First step in Spain: hospitals buildup

The Western system of health care as a single instrument for therapeutic intervention and rehabilitation, as part of the health system, is a relatively modern technology that began to be conceived in the late fifteenth century, in Europe, in line with the Renaissance thought of taking "awareness of the relationship between health problems and social conditions" which led to raise some strategies concerning "the prevention of diseases and the organization of medical care as a responsibility of the State" 1, the state was born at this time as the Spanish Universal Monarchy.

Professor Jose Antonio Maravall Casesnoves gives an account of it; he noted the relationships between the nascent modern state --that surfaced, according to him, with the Renaissance and the discovery of America-- and some problems peculiar to public health that allow to observe the role played by the Spanish crown around the resolution of health problems 2,3, such as the policy called "reduction of hospitals" caused by the increase of the needy population, the inadequate response and economic instability of hospitals, which consisted in assembling in a single place the small hospitals of a same region, according to the fusionist theories of the House of Austria (Hapsburg dynasty), which led to "reducing the number of hospitals in order to suppress the deficient ones and concentrating their income in a few, so they could, as having greater financial resources, to increase their health care capacity and to enhance their hospital work" 4.

Such so-called reduction, was actually an accumulation of hospitals that --as Recio Mir points out-- "it also allowed better control" 5, of those hospitals whose objectives would be for the Habsburgs, as Mercedes Gallent noted, "the clarity in management, profitability and efficiency" 6.

Another issue, in which the Spanish crown had to do, was the beginning of "field hospitals (ambulances) ... by Queen Isabel the Catholic during the siege of Malaga, in 1487, which were revived by his grandson Charles V during the Metz site in 1553" 7. These temporary and mobile hospitals allowed the novelty of a greater weight of hospital medical care practices, which is nothing else but a nascent medicalization 8 of them as, "since the mid-fifteenth century --as it has been observed by Mercedes Gallent-- [there is] a real 'medicalization of the hospitals' 9, all these measures that had to be matched, according to these findings, with the start of "the separation process of hospitals *sensu stricto* (in the strict sense) and workhouses" 1.

### First interpretation 

Immersed "inside politics and the complex economics of the reliefs" 10, it can be interpreted that during the fifteenth, sixteenth and seventeenth centuries began the structuring of the Western system of health care: first, with a capitalist policy of accumulating (reduction of) hospitals (what common language calls *mergers*), accumulation (concentration) of income (in finance language, it is called income *concentration*), and accumulation (concentration) of the health care capacity (what in the technical administration language is called *rationalization of resources*), "reduction in hospitals", which, besides, centralized (unified) resources in a few hands the hospital management methods and resources.

Second, with the attempt of a market segmentation by behavior characteristics and the benefits sought, the separation (specialization) of the asylums and hospitals *sensu stricto* "to which it must be added the inclusion of medical conditions and the specialization according to the various pathologies that are addressed in them" 7; third, with the incipient medicalization process of hospitals, or what is the same, the embryonic form of a hospital as an instrument of intervention and healing, because at that time -as Foucault says -"Medicine was a non-hospitable profession" 11.

## Second Step in Germany: accumulation of medical practices

After occurring the "hospitals reduction", equivalent to the idea of scale​ ​economies, conducted from 1512 in Valencia, 1532 in Segovia, 1548 in Valladolid, "1587 (in Sevilla) 12 and 1603 (in Madrid)" 1; having happened the separation attempt of hospitals *sensu stricto* and asylums, similar to the idea of ​​specialization, followed by the medicalization of hospitals; in the decadent Germanic Holy Roman Empire (now Germany), a state system of health care was generated during the seventeenth and eighteenth centuries, immersed in a system known as *medizinische Polizei* (medical police), characterized by 1) Standardization of medical and paramedical personnel (surgeons, midwives, nurses, healers, etc.) and the organization of a network with national coverage and pyramidal structure of a staff of mayors, proto-physicians, health inspectors, etc. 2) Basically urban hygienist and sanitary policy: epidemic control, fight against foci of unsanitariness and contamination ("miasmas"), sanitation of houses and streets, drainage, water and food, sanitary control of factories and hospitals, etc. and, finally 3) Families Policy: marriages, pregnancy and childbirth, breastfeeding, vaccination, orthopedics, prostitution, disability, etc 13.

This state system of health care was expressed through the "exemplary model", the "great project" and the "great construction" of Bamberg Hospital between 1787 and 1789 14, which was going to mean, first, a waiver "without excessive effort to the guidelines of the medical police, leaving it the misery domains;" which is going to imply, second, to appropriate "for itself that privileged field of experimentation that, henceforth, will be the patient's body, isolated in the ideal scenario that supposedly is the hospital; *i.e.*, that hospitals were going to complement their caring role with another (function) of a very different scientific character to the one they were hitherto entrusted" 15; and it is going to be, third, "the clinical center from which it began to radiate the reform airs of German medicine" 16.

In this sense, in the "*krankenspital* of Bamberg, opened in 1790, some important innovations appear in both the distribution of patients and the sanitary measures, and the fact of considering, next to hospital, a research and education center" 12 giving rise to the current idea that medical schools be associated with a hospital, so that students can have access to clinical teaching, with no restrictions, transcending the previous idea of ​​the hospital as a place where a physician goes to medical practice only after having studied.

### Second interpretation

It is possible to interpret that, immersed in "policies originated in the social and political grounds of the absolutist and mercantilist German state of the seventeenth and eighteenth centuries" 17 ; but especially in the late eighteenth century, in the progress in incorporating the Western system of health care, it was possible: first, to release (to disengage) the hospital of all non-medical care activities, for which the necessary civil works were carried out, which will allow it to try to intervene patients, and to try to be an instrument (a machine) for curing diseases, what Foucault calls "medical-therapeutic hospital" 11; and, second, those purposes of trying to intervene and trying to be an instrument will imply that it appropriates (acquire possession of) the privilege of being a research center on the patient's body and being a center of medical education, as a complement to its daily work: Medicine is developing as a hospital profession.

This means that, following the idea of ​ accumulation ​coming from Spain, it were collected in this case three abilities: health care or therapeutic capacity or therapeutic act, medical education or teaching ability or pedagogical act, and clinical research or epistemic capacity or epistemic act, moving to a medicalization that begins to be effective; continuing the Spanish purpose of segmenting the market for behavioral characteristics and the benefits sought, the hospital *sensu stricto* becomes real, *i.e.*, it is achieved the first specialized hospital (dedicated only to patients): the medical-therapeutic-epistemic pedagogical hospital.

## Third step in England: the hospital wards

On the other hand, after the first observations made by the Scottish physician Sir John Pringle 18 in England, about nosocomial infections caused by hospital overcrowding, "it is born the concept of modern hospital, mainly based on theoretical studies and practical experience, [which are going to be materialized in] the new buildings of *St. Bartholomew's Hospital* [within which it was considered essential to prevent the spread of infections] separation (specialization) between the buildings that surrounded the courtyard, to allow free access to air and light," of which gives account in his research Pedro Garcia Barreno 12. Specialization or separation based --as Maria de las Mercedes Insua observes-- in a "system of small pavilions, isolated from each other [to] achieve better ventilation of buildings and a specialized patients' separation" 19.

This conception of the modern hospital had several antecedents in England: first, the poor laws that can be traced since the adoption of laws to deal with the "impotent poor" in 1536, 20 the first of which was the Act of Elizabeth, issued in 1601 -commonly known as the Elizabethan Poor Law-- 21, which established "shelters for the disabled poor (the elderly and the sick) "whose "guiding principles were present in English law until almost the second half of the twentieth century" 22; and second, while in Europe the hospitals, during the Middle Ages, were associated mainly to the private orbit of a monastery or a religious order, in England they were municipal buildings (*i.e.*, originating from public sources), generally, as a result of the reforms of the Tudor, who reigned between the fifteenth and seventeenth centuries giving municipalities the care of their own poor 23.

This environment led to a space so that, in 1676, Dr. John Petty understand --based on economic reasons-- that "it is not relevant for a state to leave physicians nor patients... stranded and by their own" 24; that in 1707, the also physician Nehemiah Grew raised -based on financial reasons-- the need to regulate the rates of payments to doctors according to their experience as a way of reducing the cost of health care 25; that in 1714, the Quaker trader John Bellers understood --based on socioeconomic reasons-that it was required a plan for a national health service that included hospitals and laboratories (an idea of ​​technical accumulation), to be used as care facilities, and research and medical education centers 26; so that between 1719 and 1740 --moved by a sense of charity- hospitals were created in Westminster, Guy, St. George, and the London Hospital 27; and that founded on the mercantilist idea of ​​health as an important element of policy, "by mid-century, specialized hospitals were created" as a result of private initiative associated with cooperative activities, in other words "they were not government (state) initiatives" 25.

### Third interpretation

It can be interpreted, for the fifteenth, sixteenth and seventeenth centuries, dominated by the idea of ​​poverty as social construction, which led to "benefactors and philanthropists that structured and adjusted their assistance to the poor through an implicit identification with insecurity to thus ensure proper behavior on the part of the poor" 28 (behaviors control), a hospital architecture appears that is based on theoretical studies and practical experiences in response to control medical care needs; concern for hospital-acquired infections (risk control), which is resolved by building small isolated pavilions guaranteeing specialized separation of the sick, in a first phase; and building specialized hospitals at a later time, in line with the modern idea of ​​the division of labor, the division of functions that, is also, a segmentation by characteristics of the benefits sought; by the proposal for a regulation of rates of payments to doctors (the prelude to the monetization of care) and by the idea of ​​a plan (a target image) for a national integrated health service for hospitals and laboratories ( accumulation of technical capacity), that were possible to use them as care facilities, research and medical education.

That is, in England happened the fully-medicalized health attention (technical, specialized), which meant the incorporation in Western of the health care as a way to intervene the sick, and it was conceived the idea of ​​health care as a tool for healing, while it was given an advance to the idea of ​​planning, almost two centuries in advance.

## Four step in France: the nosological rationality

Having released the hospital from all non-medical care activity, as a new constructive step in the design of the health care system, allowing its conversion into a human body research center, and its transformation into a place of learning, in Germany, and having as background the Spanish policy of "reducing hospitals" (which originated in France "after the disposal of Parliament [in Toulouse] of 6 February 1504, in order to bring together in a single facility all small hospitals") 12 and based on the English hospital wards model, in France during the eighteenth and nineteenth centuries, surrounded by a political and economic environment in which it occurred the conversion of general medicine "into a science of the public or the things" 29, that had to be called, as Didier Fassin says, "*hygiène publique* [as well] *médecine politique*" 30, it gives full origin to the hospice hospital, to become "like an exclusively medical space" due to the "logical evolution of the nosological thought" 31; that is, "as a center exclusively dedicated to assisting the curable sick... when the social meaning of the old hospital is finally overcome by the growing importance of medical education at the patient's bedside, and to service medical science" 12 as the *krankenspital* of Bamberg in Germany was conceived. 

Such detachment --functional, spatial and formally-- from hospital hospice, in the image and likeness of Bamberg's *krankenspital*, followed a rationality founded on three pillars, as evidenced by Blandine Barret-Kriegel: assessing the health of each individual in line with medical needs, its quantification and the appearance of the population conceived as an object of medical knowledge 32, in other words, from the perspective of interpretation, it can be said that it was taken the step to a specialized technical organization that will allow to intervene the sick. 

Such *hygiene publique* or *médecine politique*, which is nothing else but a health care system that "will have the crucial role of public hygiene, with coordinating bodies of medical care, centralization of information, standardization of knowledge, and that also adopts the appearance of a hygiene learning campaign and medicalization of the population" 33, will serve as a basement of a hospital that makes it possible, on the one hand, "to standardize the conditions of teaching and practice of medicine in the country" 34.

On the other hand, it makes possible for Jean Baptiste Le Roy to express to the French Academy of Sciences in 1785, that "a hospital room, if it is allowed to express it like that, is a real machine for the treatment of the sick" 35, which recently led to Cecilia Ruiloba, who cites Le Roy from a secondary source, to the finding that the French of that time understood the hospital as "a scientific tool", as "a 'machine' that contributes to healing," meaning "'machine'... as an instrument at the service of the man (manhood) that transforms the sick into healthy", 36 on the basis of the Western dichotomy between health and disease.

However, it emerged from the eighteenth century in France, the idea "a replacement of the hospital", for which it is stated "the organization of a 'hospitalization' at home", calculating "the economic benefits taking into account that the cost of maintaining a patient is much lower for society if he (she) is maintained and nurtured at home" 37 . 

### Fourth interpretation

It is possible to interpret that, within the perfecting of the Western's health care system during the eighteenth and nineteenth centuries, it was formally consolidated the medicalized health care, which corresponds to the modern idea of ​​the division of labor, the division of functions; such space was lit by a nosological rationality (clinical), guaranteeing its objectivity, based on the assessment of the health of the individual, based on how many are the medical needs (requirements); in other words, health care is consolidated as a way of intervention in patients, seated on the conception of the population as an object of medical knowledge (bio-statistical, demographic and epidemiological) that is going to achieve (at big scale) to normalize (standardize, homogenize) the teaching and the medical practice, while it is going to operate, in a nascent way, as a (tool or machine) of scientific character (academic and epistemic), to cure diseases, which is going to allow the rising of critics to hospitals and the emergence of the idea of ​​medicine at home.

## Fifth step in the United States: the sanitizing machine

Consolidated the medicalization, guided health care by ensuring nosological rationality intervention in the sick, and that dreams to operate as a healing tool, in the United States as in Britain, the hospitals founded during the eighteenth and nineteenth centuries were not such by government initiative, but they gave rise to the idea that "from the point of view of the economy of society, it could be cheaper to give health care in an efficient and accessible way" 26.

Enlightened by this criterion, during the twentieth century, around 1938, Alphonse R. Dochez indicates the evolution of clinical records, from a very simple one, to another that reflects the combined observations from three visiting doctors, two residents, three auxiliary, ten specialists and fourteen technicians 38; which accounts a health machine, as it was dreamed in England and France, from the foundation of Bamberg on the aspirations of Spain, that was made possible by the introduction of laboratories within a hospital: bacteriology and chemistry in 1889, and X-rays in 1896 39, and with them the nursing schools, the first social workers, nutrition and dietetics services, archival procedures and business processes, which led to the rise of hospital administrators and a growing government regulatory action, especially in its funding, which ended in the current community hospital 40; *i.e.*, in the hospital as a large-scale organization that requires an administrative rationality: the managed medicine.

At the same time, there's a continuing concern that emerged in the nineteenth century for the homogenization of the teaching and practice of medicine, uneasiness that is going to be expressed in 1910 in *A Report to the Carnegie Foundation for the Advancement of Teaching*, written by Abraham Flexner with an introduction by Henri S. Pritchett.

Then, "as scientific medicine began to prevail --during the XX century-- and it became institutionalized in the hospital, the doctor became an essential element for its operation and, at the same time, the institution became increasingly indispensable for the exercise of a good medicine" 17.

### Fifth interpretation

Finally, a final interpretation is possible, during the twentieth century the hospital (and with it the current health care system) was incorporated as a large-scale organization that is an instrument for curing diseases (the French ideal of machine), with which appeared in full, a system of health care as a way of intervention for the sick and as a tool for healing diseases guided by a nosological rationality and an economic thinking that seeks efficiency and the cheapest accessibility: the final piece of the puzzle that began with hospitals buildup.

## Conclusions

Now, it is possible to interpret the path taken to the incorporation of the health care system in the West, it has been (without exception) a way contrary to the disintegration of hospitals and resources assignation (as today), because its criterion responds to a capitalist rationality for accumulating, centralizing, concentrating and gathering; which explains the crisis of many systems of health care in the Americas, caused by the disintegration of the health care and the dissemination of technical and financial resources with results contrary to the aspirations of resources rationalization that inspired recent reforms, which have been well brought to mind by Granados and Gomez, namely: first, "The need to contain escalating costs of health systems as part of the reduction in public spending of social character social" and, second, "The escalation of the production functions costs for health care" 41.

On the other hand, we can see that this road has gone through a growing specialization path (from the hospital in *sensu stricto* to the current community hospital), for which the health care system had to accumulate capacities (first, the healthcare; second, the training and the epistemic; and third, the technical), issues that were not covered by the reforms in the Americas, for the disintegration of the health care and the dissemination of technical and financial resources makes them impossible.

Finally, that when going through such path, considerations have been twofold: on the one hand, the medical techno-science (health, education, technical and medical research); on the other hand, the issues of an administrative and financial rationality; in both cases according to the logic of accumulation. However, in the reforms made in the Americas, the first question has been virtually discarded, without realizing that it is on it how the second can be reached, putting us today in front of the conviction (not desirable at all) of having to re-walk a historic road already traveled by the lack of determination and courage of Latin American elites, to fend of the own understanding without the guidance of foreign powers or foreign organizations.
